# Possible role of glutamine synthetase in the NO signaling response in root nodules by contributing to the antioxidant defenses

**DOI:** 10.3389/fpls.2013.00372

**Published:** 2013-09-19

**Authors:** Liliana Silva, Helena Carvalho

**Affiliations:** Laboratório de Biologia Molecular da Assimilação do Azoto, Instituto de Biologia Molecular e Celular, Universidade do PortoPorto, Portugal

**Keywords:** root nodules, nitrogen fixation, glutamine synthetase, tyrosine nitration, nitric oxide, *Medicago truncatula*

## Abstract

Nitric oxide (NO) is emerging as an important regulatory player in the *Rhizobium*-legume symbiosis. The occurrence of NO during several steps of the symbiotic interaction suggests an important, but yet unknown, signaling role of this molecule for root nodule formation and functioning. The identification of the molecular targets of NO is key for the assembly of the signal transduction cascade that will ultimately help to unravel NO function. We have recently shown that the key nitrogen assimilatory enzyme glutamine synthetase (GS) is a molecular target of NO in root nodules of *Medicago truncatula*, being post-translationally regulated by tyrosine nitration in relation to nitrogen fixation. In functional nodules of *M. truncatula *NO formation has been located in the bacteroid containing cells of the fixation zone, where the ammonium generated by bacterial nitrogenase is released to the plant cytosol and assimilated into the organic pools by plant GS. We propose that the NO-mediated GS post-translational inactivation is connected to nitrogenase inhibition induced by NO and is related to metabolite channeling to boost the nodule antioxidant defenses. Glutamate, a substrate for GS activity is also the precursor for the synthesis of glutathione (GSH), which is highly abundant in root nodules of several plant species and known to play a major role in the antioxidant defense participating in the ascorbate/GSH cycle. Existing evidence suggests that upon NO-mediated GS inhibition, glutamate could be channeled for the synthesis of GSH. According to this hypothesis, GS would be involved in the NO-signaling responses in root nodules and the NO-signaling events would meet the nodule metabolic pathways to provide an adaptive response to the inhibition of symbiotic nitrogen fixation by reactive nitrogen species.

## INTRODUCTION

Leguminous plants associated with symbiotic bacteria of the family *Rhizobiaceae *are able to grow under nitrogen-limiting conditions. Key to this achievement is the bacterial ability to reduce atmospheric nitrogen in a functional symbiotic interaction, in which ammonia is provided to the plant and assimilated into organic composition by the plant enzyme glutamine synthetase (GS; EC 6.3.1.2). The establishment of this symbiosis requires a constant fine-tuned signal exchange between plant and bacteria culminating with the formation of a novel organ, the root nodule, which provide an environment suitable for bacterial nitrogen fixation (Oldroyd et al., 2011). Symbiotic nitrogen fixation is of particular agricultural and ecological importance, as it constitutes one of the largest contributions to biologically available nitrogen in the biosphere. Therefore, the identification of the regulatory signaling network underlying the symbiotic interaction is of utmost importance and has been the subject of intense research (for recent reviews, see Oldroyd et al., 2011; Udvardi and Poole, 2013). In recent years, nitric oxide (NO), widely recognized as an endogenous signaling molecule, emerged as an important player in the legume–rhizobium interaction, but its mechanisms of action are still far from being understood (Besson-Bard et al., 2008; Neill et al., 2008; Meilhoc et al., 2011; Puppo et al., 2013). To unravel the signal transduction cascade and ultimately NO function, it is necessary to identify its molecular targets. We have recently shown that GS, a key enzyme for nodule functioning, is a molecular target of NO, being post-translationally regulated by tyrosine nitration in relation to active nitrogen fixation (Melo et al., 2011). In functional nodules of *Medicago truncatula *NO production has been located in the bacteroid containing cells of the nodule fixation zone (Baudouin et al., 2006), where glutamine synthetase is highly abundant. The location of the enzyme at the sites of NO production together with its position at the center of the complex matrix of nitrogen metabolism conveys an important role of the enzyme at the crossroads of signaling events. We propose that the regulation of glutamine synthetase by NO is related to metabolite channeling to boost the nodule antioxidant defenses, linking NO signaling with nitrogen metabolism. This article discusses this hypothesis in view of the existing evidence supporting a role of glutamine synthetase in the NO signaling cascade in root nodules.

## EVIDENCE FOR A SIGNALING ROLE OF NO IN THE SYMBIOTIC INTERACTION

The formation of NO and its involvement in the legume-rhizobia symbiosis has been the subject of much research in the last few years. It is now well established that the molecule is produced in root nodules and is important both for nodule development and functioning (Meilhoc et al., 2011; Wang and Ruby, 2011; Puppo et al., 2013). Nodule formation is highly complex and involves a progression of temporally and spatially regulated events, which require extensive recognition and signaling by both partners. The first signal is plant-released flavonoids and related compounds, which elicit synthesis of lipochito-oligosaccharides (Nod factors) by rhizobia. Nod factors induce cell division in the inner root cortex and the formation of a nodule primordium. In parallel, bacteria enter the root hairs via infection threads, are released to the plant cells by endocytosis and remain surrounded by a plant-derived symbiosome membrane (Oldroyd et al., 2011). As the nodule primordia continue to grow, new plant cells are continuously being infected and fully developed legume nodules contain a large central tissue harboring thousands of nitrogen fixing bacteria. The fixed nitrogen is exported as ammonium to the plant cytosol where it is assimilated into organic compounds by plant GS. In exchange for reduced nitrogen from the bacteria, the plant provides rhizobia with reduced carbon and all the essential nutrients required for bacterial metabolism. As nitrogenase is strongly inhibited by oxygen, nitrogen fixation is made possible by the microaerophilic conditions prevailing in the nodule, where the oxygen concentration is controlled by a variable-permeability barrier in the nodule parenchyma and by leghemoglobin, an oxygen-binding plant protein regulating and delivering oxygen to the infected cells (Udvardi and Poole, 2013). As such, the process of nodulation involves infection, development and metabolic processes and the signals exchanged between the two partners will encompass very different physiological contexts. NO is known to be involved in physiological processes ranging from biotic and abiotic stress responses, to normal plant growth and development (Besson-Bard et al., 2008). A number of reports document that NO is involved in the signaling network in root nodules, both at the early steps of plant–bacteria interaction and at later stages in mature nitrogen-fixing nodules, suggesting distinct roles of the molecule at different steps of the symbiosis. This subject has been reviewed comprehensively elsewhere (Meilhoc et al., 2011; Wang and Ruby, 2011; Puppo et al., 2013). Here we recapitulate very briefly the recent disclosures obtained using the model legume *Medicago truncatula *and its symbiotic partner *Sinorhizobium meliloti.*

During early steps of the *M. truncatula*–*S. meliloti* interaction, NO has been detected both at the infection sites and in the nodule primordia, suggesting an involvement of NO in both bacterial infection and nodule organogenesis. Evidence for an important role of the molecule in nodule formation was given by the finding that NO depletion resulted in a significant delay in nodule appearance and provoked the down regulation of genes involved in nodule development (del Giudice et al., 2011). In fully developed root nodules NO has been located exclusively in the infected cells and appears to be confined to the nodule fixation zone, pointing to an involvement of the molecule in root nodule metabolism (Baudouin et al., 2006; Horchani et al., 2011). A metabolic function for NO in providing a significant energy input in mature nitrogen-fixing nodules through the nitrate-NO respiration process has been recently highlighted (Horchani et al., 2011). NO has also been shown to modulate the expression of a wide number of genes both from *S. meliloti *(Meilhoc et al., 2010) and *M. truncatula *(Ferrarini et al., 2008). Many of the NO-responsive *M. truncatula* genes are involved in nodule development and functioning, with a significant number of the NO-responsive genes being involved in primary metabolism, further supporting a signaling role of NO in the nodule metabolic pathways (Ferrarini et al., 2008). More recently, NO production has also been associated with nodule senescence. Using both genetic and pharmacological approaches, it was shown that NO accumulation in aging nodules of *M. truncatula* has deleterious effects on the symbiosis by inhibiting nitrogen fixation and activating nodule senescence, whereas a decrease in NO levels leads to a delay in nodule senescence (Cam et al., 2012).

The origin of NO in plants is still not clearly understood, and in root nodules the picture is even more complex because the source of NO is probably variable at different stages of the symbiotic interaction and can arise from both symbiotic partners (Meilhoc et al., 2011). Several routes capable of yielding NO in root nodules have been described: NO synthase (NOS)-like activity converting arginine to citrulline and NO (Cueto et al., 1996; Baudouin et al., 2006; Leach et al., 2010), and nitrate reductase and the electron transfer chains from both plants and bacteria (Mesa et al., 2004; Meakin et al., 2007; Gupta et al., 2011a; Horchani et al., 2011).

Nitric oxide can signal fundamental physiological processes by changing both gene expression and protein function and a major step towards understanding the mechanisms regulated by NO during the symbiosis relies on the identification of its molecular targets. This task is made difficult, because the physiological contexts underlying discrete symbiotic stages are highly variable, ranging from infection, to development and senescence and thus the molecular targets of NO are expected to vary at different stages of the symbiotic interaction. While considerable effort is being put forward to identify the molecular targets of NO using large scale approaches, either by proteomics (Cecconi et al., 2009; Chaki et al., 2009; Lozano-Juste et al., 2011) or transcriptomics (Ferrarini et al., 2008; De Michele et al., 2009; Boscari et al., 2013), GS was identified as a molecular target of NO by a simple biochemical approach (Melo et al., 2011).

## EVIDENCE FOR A CRUCIAL ROLE OF THE NODULE ANTIOXIDANT RESPONSES IN NITROGEN FIXATION

Whilst it is now evident that NO is required for nodule functioning, paradoxically it is also clear that it is a potent inhibitor of nitrogenase activity (Trinchant and Rigaud, 1982; Kato et al., 2010). The involvement of NO in nitrogenase inactivation has been demonstrated in soybean and *Lotus* after nitrate supply (Kanayama et al., 1990; Meakin et al., 2007; Kato et al., 2010). In *Lotus japonicus*, the artificial application of the NO donor sodium nitroprusside (SNP) decreased nitrogen fixation, whereas the application of a NO scavenger (cPTIO) had the opposite effect (Shimoda et al., 2009; Kato et al., 2010). Thus, the NO concentration inside the nodule needs to be maintained at levels compatible with nitrogenase activity, but still be sufficient to achieve its signaling function. This implies a balance between NO production and detoxification. The plant antioxidant responses are therefore of crucial importance to maintain nodule functioning (Pauly et al., 2006; Becana et al., 2010; Sanchez et al., 2011). Most of the antioxidants in legume nodules are also present in other plant organs or tissues, but the concentrations in nodules are generally higher, denoting a connection between N_2_ fixation and the antioxidant response (Puppo et al., 2013). The data published to date indicate that hemoglobins (Hbs) and the GSH/ascorbate pathway constitute the chief antioxidant mechanisms in root nodules (Becana et al., 2010) and will be considered separately.

## HEMOGLOBINS

The levels of NO inside the nodule appear to be controlled by Hbs, which are able to scavenge NO, and in this way may protect nitrogenase from inactivation. In legumes, three types of Hb have been described: symbiotic Hb (Lb), non-symbiotic Hb (nsHb) and truncated Hb (trHb; Bustos-Sanmamed et al., 2011). The nsHbs are subdivided into nsHb-1s (class 1 nsHbs), which have a very high affinity for O_2_, and nsHb-2s (class 2 nsHbs), which have lower affinity for O_2_ and are similar to the sHbs (Gupta et al., 2011b). The first evidence of NO binding to Hb was given by the detection of nitroso-leghemoglobin complexes (LbNO) in nodules of soybean and Lotus (Kanayama et al., 1990; Mathieu et al., 1998; Meakin et al., 2007; Sanchez et al., 2010). Later, this NO-scavenging function has also been attributed to non-symbiotic class 1 Hbs (nsHb1) in* Lotus japonicus *(Shimoda et al., 2009) and more recently the three types of Hb were found to be expressed in nodules of *Lotus japonicus*, suggesting complementary roles of the different types of Hb for root nodule formation and/or functioning (Bustos-Sanmamed et al., 2011). Because class 1 nsHbs have an extreme affinity for O_2_, it is unlikely that they function as O_2_ transporters, stores, or sensors, therefore they have been supposed to play the role of NO scavenger in NO detoxifying pathways (Gupta et al., 2011b; Igamberdiev et al., 2011). These proteins are induced upon symbiotic infection, accumulate in nitrogen fixing nodules and their overexpression enhances symbiotic N_2_ fixation, further supporting a role in NO quenching in root nodules (Shimoda et al., 2009). An NO scavenging role has also been attributed to the flavohemoprotein Hmp of the bacterial partner (Meilhoc et al., 2010). Indeed, using *S. meliloti hmp* mutant strains and Hmp overexpressing strains, it was recently shown that this protein can modulate the levels of NO inside the nodules (Cam et al., 2012). A direct relationship between NO scavenging by Hbs and nitrogen fixation is reinforced by the fact that the over-expression of either plant ns-Hb1 in the plant partner (Nagata et al., 2008; Shimoda et al., 2009) or bacterial Hbs in the rhizobial partner (Ramirez et al., 1999; Cam et al., 2012) lead to enhanced symbiotic N_2_ fixation, whereas this process is impaired in rhizobial *hmp*^-^ mutants in *M. truncatula *(Meilhoc et al., 2010; Cam et al., 2012). All together, the available data suggest that both the plant and the bacterial Hbs are involved in the signaling responses to NO and are important for N metabolism in root nodules.

## GSH/ASCORBATE CYCLE

The GSH/ascorbate pathway provides one of the main antioxidant mechanisms in plants and several lines of evidence indicate that this pathway is a major contributor to the antioxidant defenses in nodules (reviewed in Matamoros et al., 2003; Pauly et al., 2006; Becana et al., 2010; Puppo et al., 2013). In legume root nodules there is a close positive correlation between nitrogenase activity, ascorbate and glutathione (GSH)/homoglutathione content (Dalton et al., 1993; Matamoros et al., 2003; El Msehli et al., 2011). The thiol tripeptides GSH and hGSH are known to be at high concentrations in nodules and to play key roles in both nodule formation and functioning (Frendo et al., 2005; Pauly et al., 2006; El Msehli et al., 2011). The substrates for GSH and hGSH synthesis are glutamate and cysteine and the pathway involves two ATP-dependent steps. In the first reaction, γ-glutamyl-cysteine synthetase (γ ECS; EC 6.3.2.2) catalyses the formation of γ-glutamylcysteine, and in the second reaction, glycine or β-alanine is added to the C-terminal site of γ-glutamylcysteine by GSH synthetase (GSHS; EC 6.3.2.3) or hGSH synthetase (hGSHS), respectively (Frendo et al., 1999, 2001). Recently, it was shown that GSHS and hGSHS follow a tissue-specific pattern of expression in the nodules of *M. truncatula*, pointing to a tissue-specific differential regulation of GSH and hGSH synthesis in *M. truncatula *(El Msehli et al., 2011). The importance of (h)GSH for nitrogen fixation was recently evidenced by studies in transgenic nodules with decreased or increased (h)GSH content in the nitrogen-fixing zone. These studies showed that the concentration of (h)GSH regulates nitrogen fixation efficiency and that a deficiency in (h)GSH impairs nodule growth (El Msehli et al., 2011).

Glutathione can readily react with NO to form S-nitrosoglutathione (GSNO) and may play an important role in regulating NO bioactivity. While the half-life of NO in biological systems is only a few seconds, GSNO is relatively stable and thought to function as a NO reservoir, since it can release NO or function as a transnitrosylating agent. The key enzyme regulating GSNO pools is S-nitrosoglutathione reductase (GSNOR), reducing GSNO to ultimately produce glutathione disulfide (GSSG), which can be reduced by glutathione reductase (GR) to re-enter the GSH pool and ammonia, which can be re-assimilated by GS (Liu et al., 2001; Lamotte et al., 2005).

Interestingly, it was reported that GSH is produced in response to elevated NO in roots of* M. truncatula* (Innocenti et al., 2007). As GS activity is inhibited by NO and one of its substrates, glutamate is also a substrate for (h)GSH synthesis, we proposed that upon NO-induced inhibition of GS, glutamate could be channeled to the synthesis of (h)GSH, contributing in this way, to the nodule antioxidant defenses and to the protection of nitrogenase from inactivation by NO. This aspect will be further discussed in the last section of this article.

## EVIDENCE FOR THE REGULATION OF GLUTAMINE SYNTHETASE ACTIVITY BY NO

Glutamine synthetase is abundantly present in root nodules where it plays a pivotal role in the assimilation of the ammonium released by nitrogen fixation. The enzyme catalyses the ATP-dependent condensation of ammonium with glutamate to yield glutamine, which can be directly exported from the nodules or used to synthetize asparagine, the main nitrogen export compound in indeterminate nodules (Vance, 2008). In the model legume *M. truncatula* GS is encoded by four expressed genes, two (*MtGS1a* and *MtGS1b*) encoding cytosolic isoenzymes, and two (*MtGS2a* and *MtGS2b*) encoding plastid located isoenzymes (Stanford et al., 1993; Carvalho et al., 2000a,b; Melo et al., 2003; Seabra et al., 2010), the latter of which is exclusively expressed in the seeds and is unique to *M. truncatula* and closely related species (Seabra et al., 2010). The other three GS genes are expressed in root nodules, but *MtGS1a* is highly up regulated, accounting for the production of over 90% of the total nodule GS activity, and encodes the isoenzyme responsible for the assimilation of the ammonia released by nitrogen fixation (Carvalho et al., 2000a). We have previously shown that MtGS1a is abundantly present in the infected cells of the nodule fixation zone (Carvalho et al., 2000a), coinciding with the major site of NO formation in this model species (Baudouin et al., 2006; Horchani et al., 2011). The enzyme is thus *in vivo* accessible to the oxidative effects induced by this reactive compound and it was shown to be a molecular target of NO in root nodules (Melo et al., 2011). *In vitro* studies using purified recombinant enzymes, demonstrated that the *M. truncatula* nodule enzyme MtGS1a is subjected to tyrosine nitration and that this modification provokes a total loss of enzyme activity (Melo et al., 2011). It is noteworthy that the plastid located GS isoenzyme, MtGS2a, which is also expressed in root nodules but at considerably lower levels, is also affected by NO, but by a different mechanism, cysteine nitrosylation. The finding that two isoenzymes that share a high degree of sequence homology and a remarkably conserved active site fold are differentially modified by NO, strengthens the idea that the NO signaling effects are specific under different physiological contexts. In addition to a differential sensitivity of individual GS isoenzymes to NO, the differential localization of the isoenzymes in specific organelles and/or plant tissues is likely to be implied in the NO-mediated regulation of GS activity. Future studies should address the regulation of the plastid located GS isoenzyme by S-nitrosylation. The enzyme is also expressed in the infected cells of root nodules and its expression is positively correlated with active nitrogen fixation (Melo et al., 2003). Here, we will focus on the regulation of MtGS1a by NO, because it is the *M. truncatula* GS isoenzyme responsible for the assimilation of the ammonium released by bacterial nitrogenase.

## MECHANISTIC OF MtGS1a INACTIVATION BY TYROSINE NITRATION

Protein tyrosine nitration is a post-translational modification (PTM) mediated by reactive nitrogen species (RNS), resulting from the addition of a nitro (–NO_2_) group to one of two equivalent ortho carbons in the aromatic ring of tyrosine residues (Radi, 2004). The incorporation of a nitro group (–NO_2_) into protein tyrosines can lead to profound structural and functional changes, the most common being loss of function (Radi, 2013). This PTM has been best studied in animals and it is a relatively new area of research in higher plants. A number of nitrated proteins have been identified in plants by proteomic approaches (Cecconi et al., 2009; Chaki et al., 2009; Lozano-Juste et al., 2011; Begara-Morales et al., 2013), however the functional effects of nitration on specific proteins are Known only for a few plant proteins (Lozano-Juste et al., 2011; Corpas et al., 2013) and the physiological significance of this PTM remains largely unknown. The tyrosine nitration of MtGS1a has become a good case study on how nitration of tyrosines can promote conformational changes leading to a loss of function. Furthermore, the nitration of MtGS1a in root nodules negatively correlates with active nitrogen fixation, strongly suggesting that the nitration of the enzyme is physiologically relevant for root nodule functioning, an aspect that will be further discussed in the next section.

**FIGURE 1:**
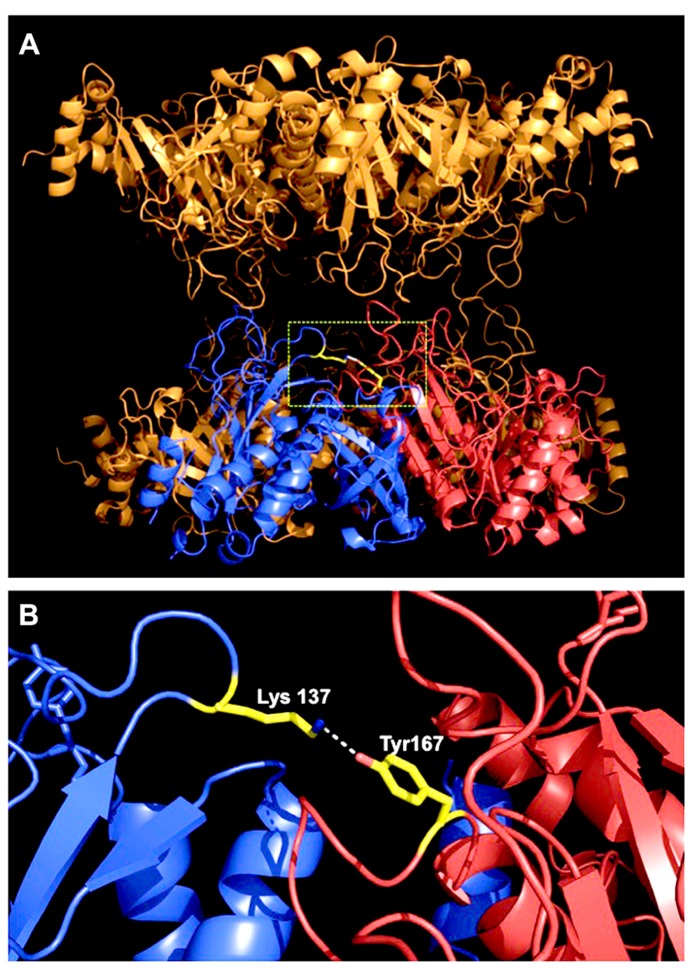
**Location of the regulatory nitration site within the three-dimensional structure of MtGS1a.** **(A)** Side-view of the MtGS1a molecule, which is a decamer composed of two stacked (face-to-face) pentameric rings, with 10 active sites formed between the C-terminal domain of one subunit and the N-terminal domain of the other subunit within a pentameric ring (Seabra et al., 2009). The position of Tyr167 is shown in yellow, in a solvent-accessible loop at the interface between two neighboring subunits, which are colored blue and red. **(B)** Arrangement of two neighboring subunits, highlighting the position of tyrosine 167 of the subunit labeled in red, close to the enzyme active site, and establishing an hydrogen bound with Lys-137 of the neighboring subunit, which is presented in blue.

Tyrosine nitration is considered a selective process, and typically only one or two of the tyrosine residues present in a protein become preferentially nitrated, depending on the structural environment (Abello et al., 2009). By site-directed mutagenesis it was shown that at least two of the 19 tyrosine residues of MtGS1a are prone to nitration, as the substitution of either Tyr 167 or Tyr 263 to phenylalanine reduced by half the protein anti-nitrotyrosine immunoreactivity. However, only mutation on Tyr 167 results in a significant reduction in the NO-mediated inhibitory effect, thus indicating that it is the relevant regulatory site (Melo et al., 2011). Since the three-dimensional structure of MtGS1a is available (Seabra et al., 2009), it was possible to enlighten the structural basis by which the nitration of Tyr167 leads to enzyme inactivation. An analysis of the structural environment of Tyr167 revealed that this residue is located in a solvent-accessible loop, close to the enzyme active site and in close proximity to a basic residue (Lys-137). In the *M. truncatula* enzyme, Tyr-167 establishes a hydrogen bond with Lys-137, and the nitration of this residue could prevent the formation of this bond, which appears to be important to maintain a correct conformation of the active site and is expected to interfere with the catalytic activity of the enzyme (**Figure 1**). Thus, the mechanism of MtGS1a inactivation by tyrosine nitration can be elucidated in structural terms. A rare example, since protein tyrosine nitration is a non-enzymatic mechanism based on free radical reactions and its selectivity for target residues in proteins is far from obvious.

## PHYSIOLOGICAL SIGNIFICANCE OF GS NITRATION FOR ROOT NODULE FUNCTIONING

Glutamine synthetase in conjunction with NADH-glutamate synthase (NADH-GOGAT, EC 1.4.1.14) operates the GOGAT cycle leading to the synthesis of glutamine and glutamate, which then serve as nitrogen donors for the biosynthesis of essentially all nitrogenous compounds. In temperate legumes, fixed nitrogen is exported from the nodules to the rest of the plant mainly as asparagine, which is synthesized by the concerted action of two additional enzymes, aspartate aminotransferase (AAT, EC 2.6.1.1) and asparagine synthetase (AS, EC 6.3.5.4). Being the first enzyme of the pathway, GS is placed in a key position to play a regulatory role in the nitrogen assimilatory pathways in nodules. The finding that it is a molecular target of NO, is thus particularly interesting. The formation of NO by plants is necessarily closely linked to nitrogen metabolism, since it is produced from inorganic (reduction of nitrate via nitrite; Horchani et al., 2011), or organic nitrogen sources like arginine via NOS-like activity (Cueto et al., 1996; Baudouin et al., 2006) and potentially polyamines, via a yet non-identified polyamine oxidation pathway (Gupta et al., 2011a; Meilhoc et al., 2011). Research in nodules of *M. truncatula* established that NO accumulation is not a by-product of symbiotic nitrogen fixation (Baudouin et al., 2006), however any of the NO generating pathways that have been described will require adequate nitrogen supply at the sites of NO production. GS is a key enzyme in nitrogen metabolism and in addition to its vital role in primary N assimilation is also crucial in N recycling in plants. Therefore, the regulation of GS by NO establishes a connection between NO signaling and N metabolism.

Nitric oxide is a strong inhibitor of nitrogenase activity, and it seems reasonable that the same signaling molecule also inhibits GS, the enzyme that uses the product of nitrogenase activity as a substrate. Indeed, in root nodules of *M. truncatula* it was shown that GS is inactivated by tyrosine nitration *in planta* and that the GS nitration status is positively correlated with the inhibition of nitrogen fixation (Melo et al., 2011). The GS nitration status was quantified *in planta* in situations where nitrogen fixation is impaired and NO is known to be produced, namely in ineffective nodules, induced either by *nifH-* or *fixJ-*rhizobial strains, as well as in nodules fed with nitrate or treated with the NO donor SNP. A direct relationship could be established between increased GS nitration, reduced nodule GS activity and reduced nitrogen fixation activity, strongly suggesting that GS is post-translationally inactivated by NO-mediated nitration in response to lower nitrogen fixation rates (Melo et al., 2011). NO concentration is expected to raise in root nodules following nitrate application (Kanayama et al., 1990; Kato et al., 2010), however in ineffective nodules NO production appears to be unaffected (Baudouin et al., 2006) and thus it seems that the regulation of GS activity by tyrosine nitration is a specific process associated with nitrogen fixation rather than a general effect resulting from increased NO levels inside the nodule.

Additional evidence for a specific regulation of GS by NO in root nodules is given by recent studies using *S. meliloti *strains carrying a mutation in the gene encoding flavohemoglobin (*hmp*), which is involved in NO degradation and leads to increased NO content inside the nodules(Meilhoc et al., 2010; Cam et al., 2012). Quantification of GS nitration in *hmp*^-^ mutant nodules revealed a considerable increase in GS nitration in relation to wild type nodules, with a concomitant decrease in GS activity (H. Carvalho, unpublished results). As it has been shown that nodules formed by the *hmp*^-^ mutant rhizobium suffer a premature senescence induced by NO (Cam et al., 2012), it is tempting to speculate that the NO-induced GS inhibition could be associated with this premature nodule senescence. This idea is supported by the finding that the application of the GS inhibitor phosphinothricin (PPT) to root nodules promotes nodule senescence (Seabra et al., 2012).

The finding that the root enzymes appear to respond differently to NO also supports a specific role of GS in the NO signaling response in root nodules. Following nitrate supply, the GS nitration status was found to be unaffected in roots but increased in root nodules (Melo et al., 2011). The total amount of nitrated proteins, which was quantified by direct ELISA using a specific anti-nitrotyrosine antibody, increases in both the roots and the nodules following nitrate supply, but GS does not appear to be among these proteins in the roots (H. Carvalho, unpublished results). It is noteworthy that in *M. truncatula* roots, GS is mainly composed of a different cytosolic isoenzyme, MtGS1b, which is largely located in the root cortex, whereas MtGS1a is confined to the root vascular tissues (Carvalho et al., 2000b). It is probable that both the formation of NO at the sites of expression of each individual GS isoenzyme and the differential sensitivity of the two isoenzymes to NO account for the differential regulation of GS in roots and root nodules. This is in agreement with the general idea that the effects of NO are not simply a consequence of the amount of NO produced but, are determined by the local environment in which NO is released and the nature of the generated RNS, which in turn will be dependent of the cellular redox state, the bioavailability of NO-generating enzyme substrates, the nature and proximity of molecular targets and of NO-metabolizing proteins.

Taken together, the available information supports a role of NO in mediating GS activity in root nodules as a function of the nitrogen fixation status, rather than this being a consequence of a general increase in NO concentration inside the nodule.

## PROPOSED MODEL FOR THE INVOLVEMENT OF GS IN THE NO SIGNALING PATHWAY IN ROOT NODULES

**FIGURE 2:**
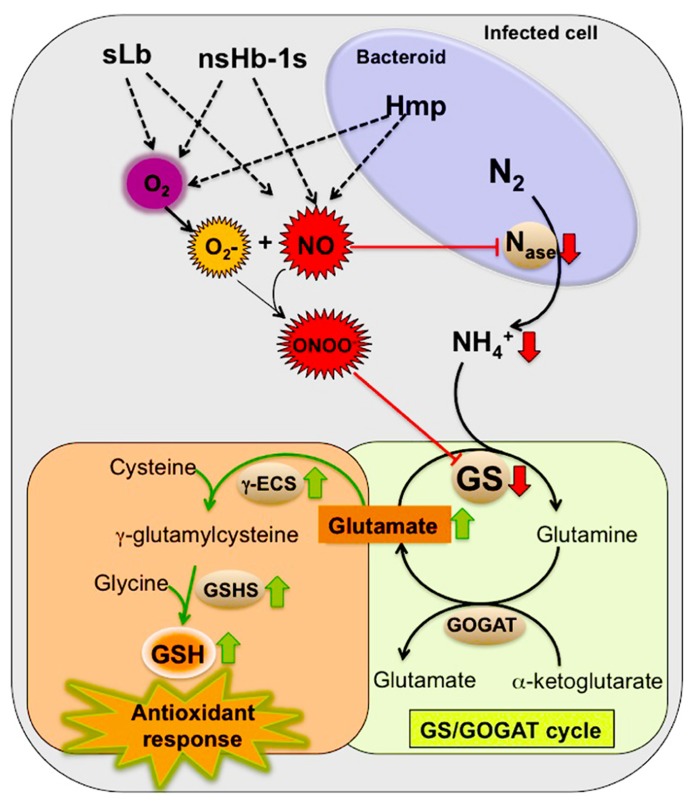
**Proposed model for the involvement of GS in the NO signaling events in root nodules by contributing to the nodule antioxidant responses.** Red arrows indicate down regulation by NO and the green arrows indicate up regulation by NO. Enzymes: nitrogenase (Nase), glutamine synthetase (GS), glutamate synthase (GOGAT), γ-glutamylcysteine synthetase (γ-ECS) and glutathione synthetase (GSHS). Hemoglobins: symbiotic leghemoglobin (sLb), class 1 non-symbiotic hemoglobins (nsHb-1s), rhizobial flavohemoprotein (Hmp).

We hypothesize that the inactivation of GS by tyrosine nitration is an NO-mediated regulatory process important for nodule functioning. In view of the overall available evidence, which was described in the previous sections, we propose a model to explain the involvement of GS in the NO signaling pathway in root nodules (**Figure 2**). According to this model, the inhibition of GS activity by tyrosine nitration would be directly related to the NO-induced nitrogenase inhibition. In view of the fact that elevated levels of NO in root nodules lead to decreased production of ammonium for GS assimilation, the enzyme would be shut down by post-translational inactivation through tyrosine nitration in response to the signal NO, the same signal that shuts down nitrogenase. This way, NO would be placed as a regulatory molecule coordinating N fixation and assimilation in root nodules.

We further propose that the NO-induced GS inhibition is involved in the nodule antioxidant response to NO and related RNS. Glutamate, a substrate for GS activity is also the precursor for the synthesis of GSH, which as described in a previous section, is known to be highly abundant in root nodules of several plant species and to play a major role in the antioxidant defense participating in the GSH/ascorbate cycle (Matamoros et al., 1999b, 2003; Becana et al., 2010). Upon NO-mediated GS inhibition, glutamate could be channeled for the synthesis of GSH contributing to neutralize the deleterious effects of RNS. This idea is supported by the finding that the synthesis of the two enzymes involved in GSH production from glutamate, γ-glutamylcysteine synthetase (γ-ECS) and GSHS is up regulated by NO in *M. truncatula*, correlating with the accumulation of the end product GSH (Innocenti et al., 2007). According to this theory, GS would be involved in the NO signaling pathway, functioning both as a sensor of increased levels of NO inside the nodules and as an activator, by forcing the N metabolic pathways to shift from primary N assimilation to the synthesis of GSH. This, in turn, would boost the nodule antioxidant responses and adjust the levels of NO inside the nodule. Since GSNO, formed by the reaction of NO with GSH, is thought to function as a mobile reservoir of NO bioactivity, GS would play an additional share in the NO signaling cascade by contributing to storage of the signaling molecule in the form of GSNO. NO release from GSNO would then be controlled by the enzyme GSNOR (Leterrier et al., 2011).

The proposed model also predicts that Hbs are important players in the process by regulating the levels of both O2 and NO, which may compete for binding sites, controlling in this way, the formation of peroxynitrite (ONOO–). Peroxynitrite is probably the main nitrating agent *in vivo* and is formed rapidly in the reaction of the superoxide anion ($O_{2}^{-}$) with NO (Abello et al., 2009; Arasimowicz-Jelonek and Floryszak-Wieczorek, 2011). As discussed before, it is documented that at least three types of Hbs have the capacity to scavenge NO, contributing in this way to modulate NO bioactivity and protecting nitrogenase from inactivation (Kanayama et al., 1990; Herold and Puppo, 2005; Meakin et al., 2007; Sanchez et al., 2010). We thus anticipate the participation from the plant side, of leghemoglobin and non-symbiotic Hb, pointing to class1 nsHb as the best candidates, and from the bacterial side the flavohemoprotein Hmp.

According to the proposed model, the NO-signaling events would meet the nodule metabolic pathways to provide an adaptive response to the inhibition of symbiotic nitrogen fixation by RNS.

## CONCLUSION

Post-translational nitration of key enzymes and the subsequent alteration of their catalytic properties may represent a new level of regulation of primary metabolism. Here we propose that the key nitrogen metabolic enzyme, glutamine synthetase is involved in the NO signaling pathways in root nodules by shifting primary N assimilation to the production of GSH in response to increased NO. For a signaling molecule to be effective, it needs to be produced quickly on demand, induce defined effects within a cell and to be removed rapidly and effectively when it is no longer required. According to the proposed hypothesis, GS would be involved in NO sensing and removal and also in NO storage by controlling GSNO pools. This mechanism would be important, on one hand to coordinate N-fixation and assimilation in the nodules and on the other hand, to boost the antioxidant defenses of the nodule in response to NO. The proposed model conveys an important role for the enzyme at the crossroads of signaling events, connecting nitrogen metabolism to NO production, storage and detoxification.

## Conflict of Interest Statement

The authors declare that the research was conducted in the absence of any commercial or financial relationships that could be construed as a potential conflict of interest.
